# Psychological distress, self-determination, and social capital associated with quality of life among retirees in Sarawak: a cross-sectional structural equation modelling analysis

**DOI:** 10.1186/s12889-026-27948-3

**Published:** 2026-06-08

**Authors:** Sivanandhan Selleyitoreea, Md Mizanur Rahman

**Affiliations:** Department of Community Medicine and Public Health, Faculty of Medicine and Health Sciences, Universiti Malaysia Sarawak (UNIMAS), 94300 Kota Samarahan, Malaysia Sarawak

**Keywords:** Quality of life, Psychological distress, Self-determination, Social capital, Retirees, Ageing, Malaysia, Structural equation modelling

## Abstract

**Background:**

Population aging is accelerating in middle-income countries; however, evidence on the psychosocial factors associated with later-life quality of life (QoL) remains limited in Southeast Asian settings. Psychological distress, self-determination, and social capital have each been linked to well-being in older populations, but few studies have examined these constructs together among retirees in geographically dispersed, multiethnic populations. This study examined the associations between psychological distress, self-determination, social capital, catastrophic health expenditure (CHE), and QoL among retirees in Sarawak, Malaysia.

**Methods:**

A cross-sectional survey was conducted among 1,410 retirees aged 60 years and above across nine districts in southern, central, and northern Sarawak. QoL was measured using the WHOQOL-BREF; psychological distress using the Depression, Anxiety, and Stress Scales-21; self-determination through basic psychological need satisfaction comprising autonomy, competence, and relatedness; and social capital using bonding and bridging indicators. CHE was assessed using health-related out-of-pocket expenditure thresholds. Partial Least Squares Structural Equation Modelling with 10,000 bootstrap resamples examined direct associations, statistical mediation pathways, and moderation effects. Hierarchical multiple regression assessed incremental variance explained by sociodemographic, health-related, and psychosocial variables.

**Results:**

The PLS-SEM model explained 56% of the variance in QoL. Self-determination showed the strongest positive association with QoL (β = 0.546, *p* < .001), followed by social capital (β = 0.352, *p* < .001). Psychological distress was negatively associated with self-determination (β = -0.379, *p* < .001) and social capital (β = -0.327, *p* < .001). Significant indirect associations were observed between psychological distress and QoL through self-determination (β = -0.207, 95% bootstrap confidence interval [BCa] CI: -0.233, -0.181) and social capital (β = -0.115, 95% BCa CI: -0.135, -0.097). CHE showed small, non-significant main direct and indirect associations with QoL. A small CHE by social capital interaction was observed for self-determination (β = -0.06, *p* = .013), requiring cautious interpretation. Hierarchical regression showed that health-related variables and CHE produced a large incremental increase in explained variance across Qol domains, while psychosocial variables added a further substantial 20–30%.

**Conclusions:**

In this cross-sectional sample, QoL was more strongly associated with psychological distress, self-determination, and social capital than with CHE. Self-determination appeared to be the strongest statistical pathway linking psychological distress with QoL. Healthy aging strategies may benefit from greater attention to mental health, autonomy, competence, relatedness, and social connectedness. Longitudinal studies are needed to clarify temporal relationships and causal mechanisms.

## Background

Population ageing is one of the major demographic and public health transitions of the 21st century. Globally, the number of people aged 60 years and above is projected to increase substantially by 2050, with the fastest growth occurring in low- and middle-income countries. Malaysia is undergoing a similar transition, with a growing proportion of older adults and increasing policy attention to healthy ageing, financial protection, and long-term care needs [1–4]. In Sarawak, these issues are particularly relevant because population ageing occurs alongside geographical dispersion, rural service access barriers, ethnic diversity, and socioeconomic inequalities.

Quality of life (QoL) in later life is a multidimensional construct that includes physical health, psychological well-being, social relationships, and environmental conditions. Although disease burden, age, income, and functional status are commonly examined in older adult QoL research, these factors may not fully explain why some retirees report better QoL than others, despite similar health or socioeconomic circumstances. Psychosocial factors may therefore be important for understanding later-life QoL, especially among retirees who may experience changes in social roles, income security, daily routines, autonomy, and community participation after leaving formal employment [5–8].

Psychological distress is one such factor. Symptoms of depression, anxiety, and stress may be associated with poorer QoL through reduced emotional well-being, lower perceived control, impaired daily functioning, and decreased social participation. For retirees, psychological distress may interact with health problems, financial concerns, and changes in family or community roles. However, psychological distress alone does not explain the psychological resources that may support a better QoL in later life [9–11].

Self-determination theory provides a useful framework for understanding internal psychological resources. Within this theory, well-being is closely linked to the satisfaction of three basic psychological needs: autonomy, competence, and relatedness. In this study, self-determination specifically refers to basic psychological need satisfaction, rather than motivation type or motivational regulation. Autonomy reflects the ability to make meaningful choices, competence reflects perceived effectiveness in managing daily life, and relatedness reflects feeling connected and valued by others. These dimensions may be particularly relevant for retirees, whose QoL may depend not only on health status but also on whether they continue to feel capable, socially connected, and able to participate meaningfully in family and community life [12–14].

Social capital represents another important psychosocial resource. It refers to resources embedded in social relationships and community networks. Bonding social capital reflects close ties with family, friends, and familiar support networks, whereas bridging social capital reflects wider connections with community groups, organisations, and institutions. Among retirees, social capital may be associated with QoL by supporting social participation, access to informal assistance, emotional support, and community belonging. In Sarawak, where communities vary by ethnicity, rurality, and access to formal services, bonding and bridging social capital may be particularly important for older adults’ well-being [15, 16].

Despite growing interest in the psychosocial determinants of later-life well-being, psychological distress, self-determination, and social capital are often examined separately. This limits the understanding of how internal psychological resources and external social resources are associated with QoL within the same model. Examining these constructs together is important because psychological distress may be linked to poorer QoL partly through lower autonomy, competence, relatedness, and weaker social connectedness. At the same time, social capital may provide contextual resources that shape how retirees experience psychological and financial stressors.

This study, therefore, examined the associations between psychological distress, self-determination, social capital, catastrophic health expenditures, and QoL among retirees in Sarawak, Malaysia. The study aimed to assess direct associations, statistical mediation pathways, and moderation effects using Partial Least Squares Structural Equation Modelling and evaluate whether psychosocial variables explained additional variance in QoL beyond sociodemographic, economic, and health-related factors.

## Methods

### Study design and setting

This cross-sectional analytical study was conducted among retirees in Sarawak, Malaysia. Sarawak was selected because it is Malaysia’s largest state by land area and has substantial geographical, ethnic, socioeconomic, and health service access diversity. Data were collected across nine districts representing three geographical zones: Southern Sarawak, comprising Bau, Kuching, Lundu, and Debak; Central Sarawak, comprising Selangau and Song; and Northern Sarawak, comprising Tatau, Miri, and Sibuti. These districts were selected to include rural, semi-urban, and peri-urban retiree populations.

### Participants and sampling

Eligible participants were retirees aged 60 years and above who had retired from public, quasi-public, or private-sector employment and were residing in the selected districts during the study period. Participants were included if they were able to provide informed consent and complete an interviewer-administered questionnaire. Individuals who were unable to provide informed consent or who were too medically unwell to complete the interview were excluded. A proportionate stratified sampling approach was used, with allocation based on the selected districts and geographical zones. The required sample size was estimated using the single-proportion formula for cross-sectional studies, with finite-population correction and adjustments for clustering and nonresponse. The calculation used an estimated older population of 353,800, an expected prevalence of 30%, a 95% confidence level, an absolute precision of 3%, and a design effect of 2.0 to account for the multistage sampling design. The estimated sample size after finite population correction was then inflated to account for an anticipated 10% nonresponse rate, resulting in a final planned sample size of 1,988 retirees. The final achieved sample was 1,410 retirees, corresponding to an overall response rate of 70.9%. District-level response rates ranged from 70.0% to 73.3%. No replacement sampling was conducted for non-respondents.

### Data collection

Data were collected using face-to-face interviewer-administered questionnaires. This approach was used because a substantial proportion of the target population had primary-level or no formal education, and interviewer-administered administration helped minimise missing data and improve comprehension. Trained research assistants administered the questionnaire in Bahasa Malaysia. Bilingual assistants provided clarification in local languages, including Iban and Bidayuh, where required. Each interview took approximately 20 to 25 min. Written informed consent was obtained from all participants before data collection.

### Measures

Quality of life was measured using the World Health Organization Quality of Life Brief Version, which assesses four domains: physical health, psychological health, social relationships, and environment. Domain scores were transformed to a 0 to 100 scale, with higher scores indicating better QoL.

Psychological distress was measured using the Depression, Anxiety, and Stress Scales (DASS-21) 21. The instrument contains three 7-item subscales measuring depression, anxiety, and stress. Items are rated on a 4-point scale from 0 to 3, and subscale scores were doubled according to the standard DASS-21 scoring procedures. In the structural model, psychological distress was represented by depression, anxiety, and stress indicators.

Self-determination was operationally defined as basic psychological need satisfaction, consistent with self-determination theory. The construct comprised autonomy, competence, and relatedness. Autonomy reflects perceived choice and volition, competence reflects perceived effectiveness in managing daily life, and relatedness reflects perceived connection and belonging. Scores were rescaled to a 0 to 100 scale, with higher scores indicating greater basic psychological need satisfaction.

Social capital was measured using bonding and bridging social capital indicators. Bonding social capital reflected close support networks, trust in routine support systems, and social network resources. Bridging social capital reflected wider community group connections, representation of retirees’ interests, responsive organisational support, and organisational assets. Items were rated on a 4-point scale, with higher scores indicating greater perceived social capital.

Catastrophic health expenditure was measured using internationally accepted budget-share and capacity-to-pay approaches. The primary CHE indicator was defined as out-of-pocket health expenditure exceeding 10% of the total household expenditure or income, consistent with financial protection monitoring approaches. A secondary CHE indicator was defined as out-of-pocket health expenditure exceeding 40% of the household capacity to pay, where capacity to pay refers to the household resources remaining after basic subsistence needs. For the structural model, CHE was entered as an observed binary variable, coded as 1 = experienced CHE and 0 = did not experience CHE. The primary SEM analysis used the 10% CHE threshold based on the one-month recall period because this indicator captured recent household-level health-related financial burdens and was consistent with the descriptive CHE estimate reported in the study.

Sociodemographic and health-related covariates included age, sex, ethnicity, religion, education level, retirement status, household income, household size, number of income earners, and self-reported doctor-diagnosed chronic diseases. Disease burden was summarised using the number of reported chronic conditions. These covariates were included in the hierarchical regression framework to assess the incremental contribution of psychosocial variables beyond sociodemographic, economic, and health-related factors.

### Translation, validation, and pilot testing

All study instruments underwent forward and backward translation, content validation, face validation, and pilot testing before the main survey. Content validity was assessed using the scale-level content validity index. Face validity was assessed using intraclass correlation coefficients. In this study, the instruments achieved S-CVI/Ave values above 0.90 across all domains, and face validity ICC values of 0.879 for single measures and 0.956 for average measures. Pilot testing was conducted among 130 eligible participants to assess the clarity, comprehension, and field-administration procedures.

### Statistical analysis

Data analysis was conducted using IBM SPSS Statistics version 29, JAMOVI version 2.6, and SmartPLS 4. Statistical significance was set at *p* < .05, using two-tailed tests.

Descriptive analysis was used to summarise sociodemographic, economic, morbidity, psychosocial, CHE, and QoL variables. Bivariate analysis was conducted to examine preliminary associations between key variables. Hierarchical multiple regression was then used to assess the incremental variance explained in QoL across three blocks: sociodemographic and economic variables in Block 1, health-related variables and CHE in Block 2, and psychosocial variables, including psychological distress, self-determination, and social capital, in Block 3.

Partial Least Squares Structural Equation Modelling was used to examine the theory-informed structural model. PLS-SEM was selected because the study aimed to assess a prediction-oriented model involving multiple latent constructs, indirect pathways, interaction terms, and non-normally distributed socioeconomic variables. Psychological distress, self-determination, social capital, and QoL were specified as reflective latent constructs or reflective higher-order constructs. CHE was entered as an observed binary variable [17].

The measurement model was assessed using indicator loadings, internal consistency reliability, composite reliability, convergent validity, and discriminant validity. Indicator loadings of 0.70 or higher were considered acceptable. Composite reliability and Cronbach’s alpha values above 0.70 indicated acceptable reliability. Average variance extracted values above 0.50 indicated convergent validity. Discriminant validity was assessed using the Fornell–Larcker criterion, cross-loadings, and the heterotrait–monotrait ratio.

The structural model was assessed using variance inflation factors for collinearity, path coefficients, bootstrapped confidence intervals, coefficient of determination, predictive relevance, and model fit indices. Bootstrapping with 10,000 resamples was used to estimate direct associations, indirect associations, and moderation effects. Mediation was interpreted as statistical mediation only because the cross-sectional design does not establish temporal ordering or causality. Moderation was examined using interaction terms between social capital and psychological distress and between social capital and CHE. Conditional associations were interpreted cautiously.

### Ethical approval

Ethical approval was obtained from the Ethics Committee of Universiti Malaysia Sarawak with the reference number UNIMAS/TNC/(PI/09-65/01 Jld.3 (67)). All participants provided written informed consent before participating in the study. The study was conducted in accordance with the Declaration of Helsinki.

## Results

### Participant characteristics

The sample comprised 1,410 retirees with a mean age of 68.58 years (SD = 7.43). The majority were female (59.2%), non-Malay Bumiputera (55.0%), and Christian (54.3%). The largest educational category was secondary school (32.3%), and 64.1% were fully retired. Heart disease (25.9%) and diabetes mellitus (22.6%) were the most prevalent chronic conditions; the mean number of chronic diseases was 1.12 (SD = 0.99). Mean total annual household income was RM 45,527.07 (SD = 29,686.88). At the 10% threshold, 18.2% of households incurred catastrophic health expenditures in the preceding month.

Self-rated overall QoL was moderate to high (M = 3.44, SD = 0.85), with domain mean percentage scores clustered between 59.86 (physical) and 61.67 (social). Bonding social capital was moderately high (mean = 3.27–3.33 on a 4-point scale), while bridging social capital was moderate (mean = 3.07–3.26). DASS-21 subscales indicated predominantly low to moderate symptom levels, although over half the sample reported frequent or chronic negative affect (35.5% “very often” and 15.1% “always”). The sociodemographic and economic characteristics of the participants are summarised in Table 1.

**Table 1:** Sociodemographic and economic characteristics of respondents (*N* = 1,410)

Variables	Frequency	(%)
Age in years (Binned)		
< 65	513	36.4
65–69	391	27.7
70–74	263	18.7
≥ 75	243	17.2
Gender		
Male	575	40.8
Female	835	59.2
Ethnicity		
Malay	358	25.4
Non-Bumi	276	19.6
Non- Malay Bumi	776	55.0
Religion		
Islam	392	27.8
Christian	765	54.3
Buddhist	209	14.8
Others (Hinduism, etc.)	44	3.1
Level of education		
No formal education	177	12.6
Primary school	404	28.7
Secondary school	456	32.3
Diploma	185	13.1
Degree and above	188	13.3
Family size		
1–2	410	29.1
3–4	689	48.9
≥ 5	311	22.1
Working status		
Retired	904	64.1
Working post-retirement	447	31.7
Others	59	4.2
Type of work		
Professional	68	4.8
Non-professional	360	25.5
Others	982	69.6
Family breadwinner		
Yes	400	28.4
No	1010	71.6
No. of earning people		
1	416	29.5
2	726	51.5
≥ 3	268	19.0

Percentages are calculated based on the total number of valid cases for each variable. The table presents frequencies and proportions for sociodemographic and economic characteristics

### Measurement model

The measurement model showed acceptable reliability and validity across the reflective latent constructs. The reliability and convergent validity results are presented in Table 2.

**Table 2:** Measurement model evaluation for reflective constructs (*N* = 1,410)

Construct	Items	Loading Range	Cronbach’s α	rho_A	CR	AVE	Inner VIF
Psychological Distress							
Depression	7	0.75–0.85	0.885	0.889	0.915	0.606	1.72
Anxiety	7	0.73–0.82	0.868	0.872	0.904	0.576	1.69
Stress	7	0.76–0.85	0.892	0.897	0.920	0.623	1.74
Self-Determination							
Autonomy	8	0.75–0.83	0.890	0.894	0.918	0.584	1.58
Competence	8	0.72–0.81	0.876	0.880	0.909	0.563	1.55
Relatedness	8	0.75–0.84	0.894	0.899	0.921	0.602	1.58
Social Capital							
Bonding SC	21	0.71–0.84	0.905	0.910	0.926	0.592	1.38
Bridging SC	21	0.70–0.83	0.892	0.897	0.918	0.571	1.38
Quality of Life							
Physical	7	0.70–0.82	0.862	0.867	0.901	0.571	1.63
Psychological	6	0.75–0.83	0.870	0.875	0.907	0.622	1.59
Social	3	0.81–0.87	0.821	0.826	0.884	0.718	1.44
Environment	8	0.69–0.80	0.858	0.862	0.896	0.523	1.61

Higher-order constructs were represented by their respective first-order dimensions

One environmental item had a borderline loading of 0.69 and was retained because of its content relevance

*CR* composite reliability, *AVE *average variance extracted, *VIF *variance inflation factor, *SC *social capital

Indicator loadings were generally above the recommended threshold of 0.70, except for one environment item with a loading of 0.69 that was retained because of content relevance. Cronbach’s alpha values ranged from 0.821 to 0.905, rho_A values from 0.826 to 0.910, and composite reliability values from 0.884 to 0.926, indicating acceptable internal consistency reliability. Average variance extracted values ranged from 0.523 to 0.718, supporting convergent validity. Discriminant validity was assessed using the Fornell–Larcker criterion and HTMT values. The discriminant validity results are presented in Table 3.

**Table 3:** Discriminant validity assessment using the Fornell-Larcker criterion and HTMT values

	Psych. Distress	Self-Determination	Social Capital	QoL
Psychological Distress	0.781			
Self-Determination	−0.480	0.762		
Social Capital	−0.440	0.520	0.755	
Quality of Life	−0.590	0.570	0.490	0.794

Diagonal values represent the square root of AVE, and off-diagonal values represent inter-construct correlations based on the Fornell-Larcker criterion. HTMT values were: Psychological distress-SDT = 0.560; Psychological distress-SC = 0.500; Psychological distress-QoL = 0.680; SDT-SC = 0.610; SDT-QoL = 0.720; SC-QoL = 0.580. All HTMT values were below 0.85

*SDT* self-determination, *SC *social capital, *QoL *quality of life

The square root of AVE for each construct exceeded its correlations with other constructs. All HTMT values were below 0.85, supporting the discriminant validity.

### Structural model

The structural model explained 56% of the variance in QoL (R² = 0.56, adjusted R² = 0.56), 14% of the variance in self-determination (R² = 0.14), and 11% of the variance in social capital (R² = 0.11). Collinearity was not problematic, with VIF values below accepted thresholds. The mediation model showed an SRMR of 0.10, which was interpreted as a borderline approximate fit. Therefore, the structural model was interpreted using theoretical plausibility, measurement quality, collinearity diagnostics, path coefficients, explained variance, and predictive relevance rather than global fit alone. Predictive relevance was supported by Q²predict values of 0.278 for QoL, 0.141 for self-determination, and 0.105 for social capital. The direct, indirect, and total associations in the structural model are presented in Table 4.

**Table 4:** Direct, indirect, and total associations in the structural model

Path	β	SE	95% BCa CI	*p*
Direct associations				
Psych. Distress → Self-determination	−0.379	0.023	−0.417, − 0.340	< 0.001
Psych. Distress → Social capital	−0.327	0.024	−0.366, − 0.287	< 0.001
Self-determination → QoL	0.546	0.016	0.519, 0.572	< 0.001
Social capital → QoL	0.352	0.017	0.323, 0.380	< 0.001
CHE → Self-determination	−0.021	0.025	−0.062, 0.021	> 0.05
CHE → Social capital	−0.027	0.025	−0.068, 0.015	> 0.05
Indirect associations				
Psych. Distress → SDT → QoL	−0.207	0.016	−0.233, − 0.181	< 0.001
Psych. Distress → SC → QoL	−0.115	0.012	−0.135, − 0.097	< 0.001
CHE → SDT → QoL	−0.011	0.014	−0.034, 0.011	> 0.05
CHE → SC → QoL	−0.009	0.009	−0.024, 0.005	> 0.05
Total associations				
Psych. Distress → QoL	−0.322	0.019	−0.354, − 0.290	< 0.001
CHE → QoL	−0.021	0.018	−0.050, 0.010	> 0.05

Direct, indirect, and total associations were estimated using 10,000 bootstrap resamples with bias-corrected 95% confidence intervals

Self-determination refers to basic psychological need satisfaction, comprising autonomy, competence, and relatedness

*SDT* self-determination, *SC *social capital, *CHE *catastrophic health expenditure, *QoL *quality of life

Self-determination showed the strongest positive association with QoL (β = 0.546, *p* < .001), followed by social capital (β = 0.352, *p* < .001). Psychological distress was negatively associated with self-determination (β = -0.379, *p* < .001) and social capital (β = -0.327, *p* < .001). Significant indirect associations were observed between psychological distress and QoL through self-determination (β = -0.207, 95% BCa CI: -0.233, -0.181) and through social capital (β = -0.115, 95% BCa CI: -0.135, -0.097).

CHE showed small and non-significant direct associations with self-determination (β = -0.021, *p* > .05) and social capital (β = -0.027, *p* > .05). The indirect associations between CHE and QoL through self-determination and social capital were also non-significant. The total association between CHE and QoL was small and non-significant (β = -0.021, *p* > .05). These findings indicate that, in the adjusted structural model, CHE did not show an association with QoL comparable to that of the psychosocial variables.

### Moderation analysis

Moderation analysis was conducted to examine whether social capital modified the associations between psychological distress and self-determination and between CHE and self-determination. The moderation results are presented in Table 5.

**Table 5:** Moderation analysis of social capital in the structural model

Interaction pathway	β	SE	t	*p*
SC × Psych. Distress→ SDT	−0.01	0.02	0.33	0.370
SC × CHE → SDT	−0.06	0.03	2.22	0.013

Moderation analysis assessed whether social capital modified the associations between psychological distress and self-determination and between CHE and self-determination

Self-determination refers to basic psychological need satisfaction

*SC* social capital, *CHE *catastrophic health expenditure

Social capital did not significantly moderate the association between psychological distress and self-determination (β = -0.01, *p* = .370). The interaction between social capital and CHE was statistically significant but small (β = -0.06, *p* = .013). Simple slope analysis suggested that the negative association between CHE and self-determination was more evident at higher levels of social capital than at mean or lower levels of social capital. This interaction should be interpreted cautiously because most CHE-related main and indirect pathways were not statistically significant.

### Hierarchical regression

Hierarchical regression was conducted to assess the incremental variance explained by sociodemographic, health-related, and psychosocial variables. The incremental variance explained across the hierarchical regression models is shown in Table 6.

**Table 6:** Incremental variance explained across hierarchical regression models (*N* = 1,410)

Domain	Block 1 *R*² (Sociodem & economic)	Block 2 ΔR² (+ Health and CHE)	Block 3 ΔR² (+ Psychosocial)	Final *R*²
Physical	0.02	0.34	0.20	0.56
Psychological	0.02	0.34	0.24	0.60
Social	0.02	0.34	0.24	0.60
Environmental	0.02	0.34	0.23	0.59
Overall	0.02	0.34	0.30	0.66

Block 1 included sociodemographic and economic variables. Block 2 added health-related variables and catastrophic health expenditures. Block 3 added psychosocial variables, including psychological distress, self-determination, and social capital

*QoL* quality of life, *CHE *catastrophic health expenditure

Health-related variables and CHE accounted for a large incremental increase in explained variance (ΔR² = 0.34 across domains), while psychosocial variables added a further 20% to 30% of explained variance across QoL outcomes. In the final models, the total explained variance ranged from 56% for the physical QoL domain to 66% for overall QoL. In the final overall QoL model, autonomy (β = 0.23), relatedness (β = 0.24), and competence (β = 0.17) were the strongest positive psychosocial predictors. In contrast, depression (β = -0.16), stress (β = -0.12), and anxiety (β = -0.08) were negatively associated with QoL. Sociodemographic variables showed weaker associations in the final models after health-related and psychosocial variables were included.

## Discussion

### Principal findings

This cross-sectional study examined the associations between psychological distress, self-determination, social capital, catastrophic health expenditures, and quality of life among retirees in Sarawak. The findings indicate that quality of life was most strongly associated with self-determination, followed by social capital and psychological distress. Self-determination, operationalised as basic psychological need satisfaction involving autonomy, competence, and relatedness, showed the strongest positive association with quality of life. Psychological distress was negatively associated with self-determination and social capital, and significant indirect associations were observed between psychological distress and quality of life through both self-determination and social capital.

Because this study was cross-sectional, these indirect associations should be interpreted as theory-informed statistical pathways rather than evidence of temporal or causal mechanisms. Cross-sectional mediation can be useful for testing theoretically specified models; however, it cannot establish the time sequence required for causal inference [18, 19].

In contrast, catastrophic health expenditures showed small, non-significant main direct and indirect associations with quality of life in the adjusted structural model. A small interaction between catastrophic health expenditures and social capital was observed for self-determination; however, this finding should be interpreted cautiously because most CHE-related pathways were not statistically significant. Overall, the findings suggest that psychosocial factors, particularly psychological distress and self-determination, were more consistently associated with quality of life than CHE in this sample.

### Self-determination and quality of life

The strongest finding of this study was a positive association between self-determination and quality of life. In this manuscript, self-determination refers specifically to basic psychological need satisfaction, comprising autonomy, competence, and relatedness. This distinction is important because the instrument used in the study assessed satisfaction of basic psychological needs rather than motivation type or motivational regulation.

The findings are consistent with self-determination theory, which proposes that well-being is closely linked to the satisfaction of autonomy, competence, and relatedness [20]. Evidence from later-life research also suggests that basic psychological need satisfaction is positively associated with well-being indicators in older populations [12]. Among retirees, these needs may be particularly relevant. Autonomy may reflect the ability to make meaningful choices about daily routines, healthcare decisions, finances, and family roles. Competence may reflect confidence in managing health, household responsibilities, and social participation. Relatedness may reflect feeling valued, connected, and supported by family, peers, and the wider community [21, 22].

The association between self-determination and quality of life may therefore reflect the importance of perceived agency and meaningful participation in later life. Retirement can alter occupational identity, income security, social routines, and decision-making roles within the household. Retirees who continue to feel capable, connected, and able to make meaningful choices may report a better quality of life across physical, psychological, social, and environmental domains [23, 24]. However, because of the cross-sectional design, these findings should be interpreted as associations rather than evidence that self-determination causes better quality of life.

### Psychological distress and self-determination

Psychological distress was negatively associated with both self-determination and social capital. This finding suggests that retirees with higher symptoms of depression, anxiety, and stress were more likely to report lower autonomy, competence, relatedness, and social connectedness. The significant indirect association between psychological distress and quality of life through self-determination indicates that basic psychological need satisfaction may be an important statistical pathway linking distress to a poorer quality of life [13, 25, 26].

This pathway is theoretically plausible. Depression may be associated with reduced motivation, hopelessness, and withdrawal from meaningful activities. Anxiety may reduce perceived control and confidence in managing health, finances, or social interactions. Stress may reduce emotional regulation and increase perceived burden in daily life. Together, these symptoms may be associated with lower perceived autonomy, reduced competence, and weaker relatedness. Lower need satisfaction may then be reflected in a poorer quality of life [12, 27].

However, this mediation should be interpreted cautiously. The analysis demonstrates a statistically significant indirect association but does not establish temporal ordering. It is also possible that a poorer quality of life contributes to psychological distress or that the relationship is bidirectional. Longitudinal studies are needed to determine whether psychological distress precedes lower self-determination and poorer quality of life, or whether these factors reinforce one another over time [18, 19, 28, 29].

### Social capital and quality of life

Social capital was positively associated with quality of life and also carried part of the indirect association between psychological distress and quality of life. This finding supports the relevance of social connectedness to later-life well-being. Bonding social capital, such as close family, friends, and familiar support networks, may provide emotional support, practical assistance, and a sense of belonging. Bridging social capital, such as connections with community groups, organisations, and institutions, may support wider participation, access to information, and engagement beyond the household [15, 30–33].

In Sarawak, social capital may be particularly important because older adults live across diverse geographical and cultural settings. In rural or semi-urban communities, informal networks may help retirees access support when formal services are distant or limited. At the same time, bridging ties through community organisations or older-person activity centres may provide opportunities for social participation and reduce isolation.

Nevertheless, social capital should not be interpreted as uniformly protective. The current study measured bonding and bridging social capital as general resources but did not distinguish between supportive and burdensome relationships. Social relationships may provide support, but they may also carry expectations, obligations, or social comparison. Future studies should separate positive social support from negative social interaction, perceived obligation, and relational burden to better understand how social capital operates among retirees in collectivist and multiethnic settings.

### Catastrophic health expenditure and quality of life

Catastrophic health expenditure showed small, non-significant main associations with self-determination, social capital, and quality of life in the adjusted structural model. This suggests that, after accounting for psychological distress, self-determination, and social capital, CHE did not show an independent association with quality of life comparable to the psychosocial variables. This does not mean that health-related financial burden is unimportant. Rather, in this model, its association with quality of life appeared weaker than the associations observed for psychosocial factors.

Several explanations are possible. First, the use of a binary CHE indicator may not fully capture the intensity, duration, or subjective experience of financial strain. CHE is commonly used as a financial protection indicator in universal health coverage monitoring. SDG indicator 3.8.2 tracks catastrophic health spending using thresholds such as out-of-pocket health spending exceeding 10% or 25% of total household expenditure or income, while the capacity-to-pay approach has also been widely used to assess whether out-of-pocket health spending exceeds household resources available after subsistence needs. Some retirees may experience financial stress even below the CHE threshold, while others may cross the threshold but have family support, savings, pensions, or public assistance that reduces its perceived effect on quality of life [34–37].

Second, Malaysia’s public healthcare system may reduce the direct quality-of-life consequences of out-of-pocket expenditures for some retirees. Third, the effect of the financial burden may operate through unmeasured pathways, such as perceived financial insecurity, delayed care seeking, debt, or caregiving burden [38].

The interaction between CHE and social capital was statistically significant but small. The pattern suggested that the negative association between CHE and self-determination was more evident at higher levels of social capital. One possible explanation is that, in closely connected communities, financial strain may become more socially visible or may be accompanied by perceived obligations to reciprocate support. However, this interpretation is speculative because the study did not directly measure shame, obligation, social comparison, or negative social interactions. Therefore, this interaction should be treated as an exploratory finding requiring further qualitative and longitudinal investigation.

### Sociodemographic, health-related, and psychosocial factors

The hierarchical regression findings showed that health-related variables and CHE accounted for a large incremental increase in explained variance, while psychosocial variables added further explanatory value across quality-of-life outcomes. In the final models, autonomy, relatedness, competence, depression, stress, and anxiety remained important individual predictors of quality of life. This suggests that both health-related and psychosocial factors are relevant, but the psychosocial indicators provided additional explanatory value beyond sociodemographic and health-related characteristics.

These findings should not be interpreted to mean that sociodemographic or biomedical factors are unimportant. Age, education, income, morbidity, and household structure remain important public health considerations. Recent quality-of-life research has shown that health-related and sociodemographic factors, including body mass index, long-term conditions, sex, and demographic characteristics, may be associated with health-related quality of life in general and in older adult populations [39–41]. However, the present findings suggest that psychosocial factors may help explain variation in quality of life that is not captured by demographic and disease variables alone. For healthy aging policy, this supports the need to consider mental health, perceived autonomy, competence, relatedness, and social connectedness alongside conventional clinical and socioeconomic indicators.

### Public health and policy implications

The findings have several implications for healthy aging strategies in Sarawak and similar middle-income settings. First, psychological distress among retirees should be recognised as an important public health concern. Screening for depression, anxiety, and stress in primary care and community-based older person programmes may help identify retirees who are at risk of poorer quality of life.

Second, programmes for retirees should not focus only on disease management. Interventions that support autonomy, competence, and relatedness may be relevant. Examples include shared decision-making in healthcare, self-management support for chronic diseases, skills-based community activities, peer support groups, and meaningful roles for older adults in family and community settings.

Third, social capital should be strengthened carefully. Community participation, older person activity centres, neighbourhood support, and intergenerational programmes may support quality of life. However, social programmes should avoid assuming that denser social networks are always beneficial. In some circumstances, social relationships may also involve obligation or burden, particularly when financial strain is present.

Finally, financial protection remains important, even though CHE was not the strongest factor in the adjusted structural model. Policies that reduce out-of-pocket expenditures, improve access to affordable care, and support older adults facing financial hardship remain relevant. The findings suggest that financial protection may be most useful when combined with mental health and psychosocial support.

### Strengths and limitations

This study has several strengths. It used a large sample of 1,410 retirees across nine districts in Sarawak, covering southern, central, and northern zones. The study also examined quality of life using a multidimensional framework and integrated psychological distress, self-determination, social capital, and CHE into one structural model. The use of PLS-SEM allowed assessment of direct associations, indirect associations, and moderation effects, while hierarchical regression provided complementary evidence on incremental variance explained across quality-of-life domains.

This study has several limitations must be acknowledged. First, the cross-sectional design prevents conclusions about causality or temporal order. The mediation findings represent statistical indirect associations only and should not be interpreted as evidence of the causal mechanisms. Second, the study relied on self-reported information, including morbidity, income, expenditure, psychological distress, and social capital, which may be affected by recall bias or social desirability bias. Third, the CHE indicator was binary and may not capture the full range of financial strain, subjective financial insecurity, debt, or delayed care seeking.

Fourth, although the sample was large and covered multiple districts, the findings may not be generalisable to all retirees in Sarawak or Malaysia, especially those in districts not included in the sampling frame. Fifth, some potentially important confounders were not measured, including cognitive function, functional disability, health literacy, personality traits, caregiving burden, pension adequacy, and quality of family relationships. Sixth, the social capital measure did not distinguish between supportive and burdensome dimensions of social relationships. Finally, because all variables were collected using the same survey method, common-method bias cannot be fully excluded.

## Discussion summary

In summary, this study found that quality of life among retirees in Sarawak was more consistently associated with psychological distress, self-determination, and social capital than with CHE in the adjusted structural model. Self-determination, operationalised as autonomy, competence, and relatedness need satisfaction, showed the strongest association with quality of life and represented an important statistical pathway linking psychological distress with quality of life. CHE showed limited main associations, although a small interaction with social capital was observed and should be interpreted cautiously. These findings suggest that healthy aging strategies may benefit from integrating mental health support, autonomy-supportive care, competence-building activities, and socially meaningful community participation. Longitudinal and intervention studies are needed to clarify causal pathways and identify effective strategies for improving the quality of life among retirees.

## Conclusions

In this cross-sectional study of retirees in Sarawak, quality of life was more consistently associated with psychological distress, self-determination, and social capital than with catastrophic health expenditure in the adjusted structural model. Self-determination, operationalised as basic psychological need satisfaction involving autonomy, competence, and relatedness, showed the strongest positive association with quality of life and represented an important statistical pathway linking psychological distress with quality of life. Social capital was also positively associated with quality of life, although its interaction with catastrophic health expenditure should be interpreted cautiously because most CHE-related pathways were not statistically significant.

These findings suggest that healthy aging strategies in Sarawak and similar middle-income settings may benefit from greater attention to the psychosocial dimensions of later-life well-being, including mental health, perceived autonomy, competence, relatedness, and meaningful social connectedness. Although financial protection remains important, the present findings indicate that QoL among retirees cannot be understood through financial or biomedical indicators alone. Longitudinal and intervention studies are needed to clarify temporal relationships, test causal mechanisms, and identify effective strategies for improving the QoL of retirees.

## Data Availability

The datasets generated and analysed during the current study are available from the corresponding author on reasonable request.

## Abbreviations

AVE: Average variance extracted
BCa CI: Bias-corrected and accelerated confidence interval
BPNSFS: Basic psychological need satisfaction and frustration scale
CHE: Catastrophic health expenditure
CR: Composite reliability
DASS-21: Depression, anxiety and stress scales – 21 items
HTMT: Heterotrait-monotrait ratio
PLS-SEM: Partial Least Squares Structural Equation Modelling
QoL: Quality of life
SDT: Self-determination theory
SRMR: Standardised root mean square residual
VIF: Variance inflation factor
WHOQOL-BREF: World Health Organization Quality of Life – Brief Version
